# Successful cholangioscopy-guided biopsy using a novel thin cholangioscope under balloon enteroscopy in a patient with Roux-en-Y gastrectomy

**DOI:** 10.1055/a-2271-7050

**Published:** 2024-03-14

**Authors:** Yuki Tanisaka, Masafumi Mizuide, Akashi Fujita, Takahiro Shin, Kei Sugimoto, Ryuhei Jinushi, Shomei Ryozawa

**Affiliations:** 1 183786 Gastroenterology, Saitama Medical University International Medical Center, Hidaka, Japan

Peroral cholangioscopy (POCS) is beneficial not only for direct visualization of intraductal bile duct lesions but also for biopsy under direct cholangioscopic view 1 2 . However, the concept of POCS and cholangioscopy-guided biopsy by means of balloon enteroscopy is problematic. This is because cholangioscopes are approximately 10 Fr in diameter and cannot pass through the forceps channel of the balloon enteroscope. We report a successful cholangioscopy-guided biopsy using a novel thin cholangioscope with balloon enteroscopy in a patient with Roux-en-Y gastrectomy.S

A 68-year-old woman had undergone total gastrectomy with Roux-en-Y for gastric cancer 2 years earlier. On referral, magnetic resonance imaging revealed stones and biliary stricture in the common bile duct ( Fig. 1 ). Therefore, endoscopic retrograde cholangiopancreatography (ERCP) was performed, using a short-type single-balloon enteroscope (SIF-H290, working length 152 cm, channel diameter 3.2 mm; Olympus, Japan) 3 4 , and POCS was performed using a thin cholangioscope (eyeMax; Micro-Tech, China) of length 219 cm and diameter 9 Fr ( Fig. 2 ) 5 ( Video 1 ).

**Fig. 1:**
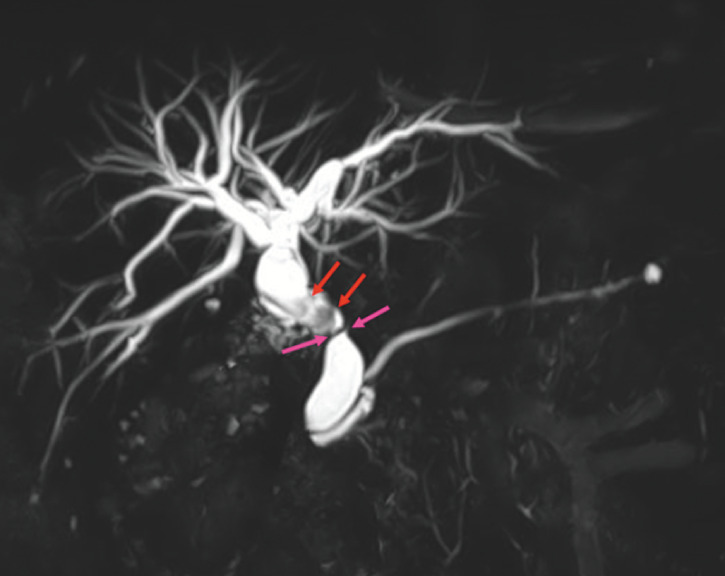
Magnetic resonance imaging (MRI) revealed stones (red arrows) and biliary stricture (pink arrows) in the common bile duct.

**Fig. 2:**
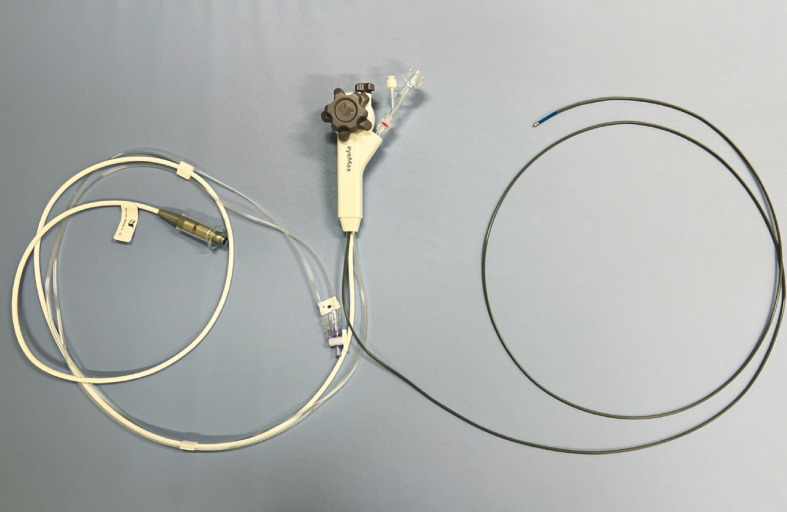
Thin cholangioscope (eyeMax; Micro-Tech, China), length 219 cm, diameter 9 Fr.

Successful cholangioscopy-guided biopsy using a novel thin cholangioscope and balloon enteroscopy in a patient with Roux-en-Y anatomy.Video 1

Cholangiography revealed bile duct stones and biliary stricture in the common bile duct ( Fig. 3 ). Subsequently, POCS was performed using the thin cholangioscope and showed no malignant findings at the stricture ( Fig. 4 **a** ), and cholangioscopy-guided biopsy was done to rule out malignancy ( Fig. 4 **b,c** ). POCS revealed bile duct stones above the stricture ( Fig. 4 **d** ). We dilated the stricture using an 8-mm dilation balloon catheter (REN; Kaneka, Osaka, Japan), and this was followed by complete stone extraction ( Fig. 5 ). Histopathological findings showed no malignancy from the stricture.

**Fig. 3:**
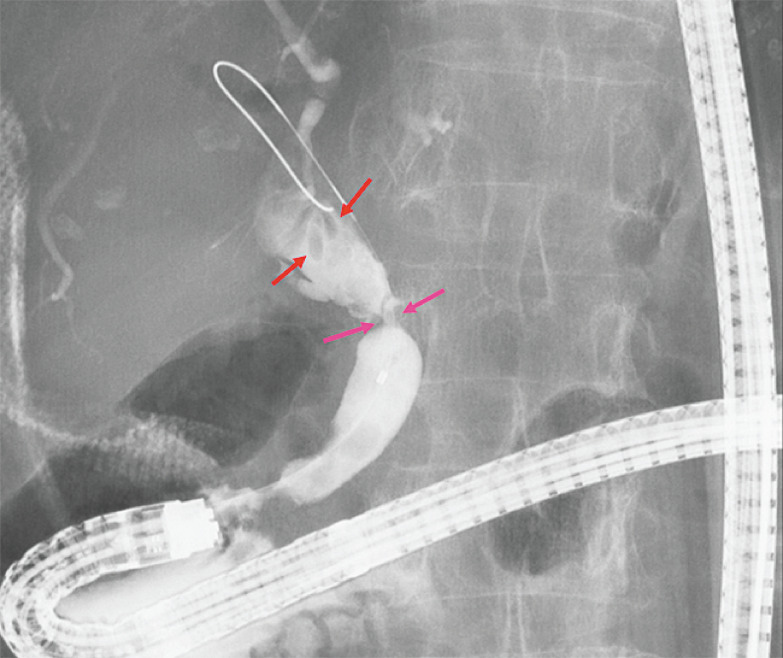
Cholangiography revealed bile duct stones (red arrows) and biliary stricture (pink arrows) in the common bile duct.

**Fig. 4:**
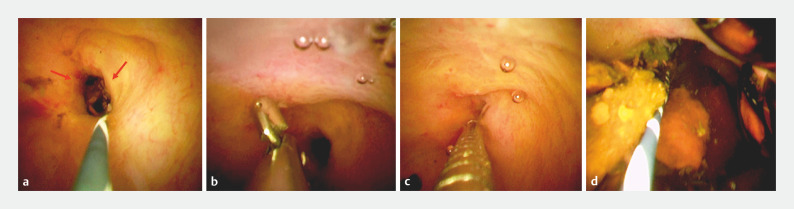
Cholangioscopy findings. **a** No malignancy was seen at the stricture (red arrows). **b,c** Cholangioscopy-guided biopsy was performed to rule out malignancy. **d** Cholangioscopy revealed bile duct stones above the stricture.

**Fig. 5:**
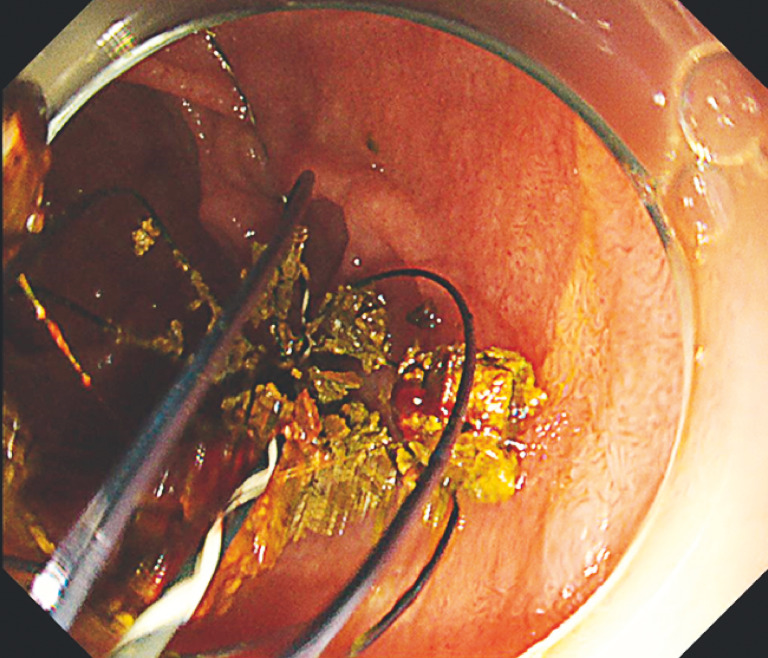
Endoscopic view of successful stone extraction.

The thin cholangioscope proved effective not only for bile duct inspection, but also for cholangioscopy-guided biopsy, even via balloon enteroscopy. This is the first cholangioscope to be used for cholangioscopy-guided biopsy via balloon enteroscopy. This novel thin cholangioscope could potentially improve the diagnostic yield in cases such as that described here.

Endoscopy_UCTN_Code_TTT_1AR_2AD

## Notes

Endoscopy E-Videos https://eref.thieme.de/e-videos
*E-Videos* is an open access online section of the journal *Endoscopy* , reporting on interesting cases and new techniques in gastroenterological endoscopy. All papers include a high-quality video and are published with a Creative Commons CC-BY license. Endoscopy E-Videos qualify for HINARI discounts and waivers and eligibility is automatically checked during the submission process. We grant 100% waivers to articles whose corresponding authors are based in Group A countries and 50% waivers to those who are based in Group B countries as classified by Research4Life (see: https://www.research4life.org/access/eligibility/ ). This section has its own submission website at https://mc.manuscriptcentral.com/e-videos .
